# Virus Infections Incite Pain Hypersensitivity by Inducing Indoleamine 2,3 Dioxygenase

**DOI:** 10.1371/journal.ppat.1005615

**Published:** 2016-05-11

**Authors:** Lei Huang, Rong Ou, Guilherme Rabelo de Souza, Thiago M. Cunha, Henrique Lemos, Eslam Mohamed, Lingqian Li, Gabriela Pacholczyk, Janice Randall, David H. Munn, Andrew L. Mellor

**Affiliations:** 1 Cancer Immunology, Inflammation and Tolerance Program, Cancer Center, Augusta University, Augusta, Georgia, United States of America; 2 Department of Pharmacology, Ribeirao Preto Medical School, University of São Paulo, Ribeirão Preto, São Paulo, Brazil; Icahn School of Medicine at Mount Sinai, UNITED STATES

## Abstract

Increased pain sensitivity is a comorbidity associated with many clinical diseases, though the underlying causes are poorly understood. Recently, chronic pain hypersensitivity in rodents treated to induce chronic inflammation in peripheral tissues was linked to enhanced tryptophan catabolism in brain mediated by indoleamine 2,3 dioxygenase (IDO). Here we show that acute influenza A virus (IAV) and chronic murine leukemia retrovirus (MuLV) infections, which stimulate robust IDO expression in lungs and lymphoid tissues, induced acute or chronic pain hypersensitivity, respectively. In contrast, virus-induced pain hypersensitivity did not manifest in mice lacking intact IDO1 genes. Spleen IDO activity increased markedly as MuLV infections progressed, while IDO1 expression was not elevated significantly in brain or spinal cord (CNS) tissues. Moreover, kynurenine (Kyn), a tryptophan catabolite made by cells expressing IDO, incited pain hypersensitivity in uninfected IDO1-deficient mice and Kyn potentiated pain hypersensitivity due to MuLV infection. MuLV infection stimulated selective IDO expression by a discreet population of spleen cells expressing both B cell (CD19) and dendritic cell (CD11c) markers (CD19^+^ DCs). CD19^+^ DCs were more susceptible to MuLV infection than B cells or conventional (CD19^neg^) DCs, proliferated faster than B cells from early stages of MuLV infection and exhibited mature antigen presenting cell (APC) phenotypes, unlike conventional (CD19^neg^) DCs. Moreover, interactions with CD4 T cells were necessary to sustain functional IDO expression by CD19^+^ DCs *in vitro* and *in vivo*. Splenocytes from MuLV-infected IDO1-sufficient mice induced pain hypersensitivity in uninfected IDO1-deficient recipient mice, while selective *in vivo* depletion of DCs alleviated pain hypersensitivity in MuLV-infected IDO1-sufficient mice and led to rapid reduction in splenomegaly, a hallmark of MuLV immune pathogenesis. These findings reveal critical roles for CD19^+^ DCs expressing IDO in host responses to MuLV infection that enhance pain hypersensitivity and cause immune pathology. Collectively, our findings support the hypothesis elevated IDO activity in non-CNS due to virus infections causes pain hypersensitivity mediated by Kyn. Previously unappreciated links between host immune responses to virus infections and pain sensitivity suggest that IDO inhibitors may alleviate heightened pain sensitivity during infections.

## Author Summary

Chronic pain is a factor in diseases that afflict many people, yet the underlying causes of pain are poorly understood. Here we assess the effects of virus infections on pain sensitivity in mice. Infecting mice with two different viruses, influenza and mouse leukemia virus (MuLV) increased pain sensitivity. Influenza infection caused transient increase in pain sensitivity, which returned to normal levels after infections were cleared. However persistent MuLV infections caused sustained increase in pain sensitivity. Virus-induced pain sensitivity was reduced substantially in mice lacking the enzyme indoleamine 2,3 dioxygenase (IDO), which degrades the amino acid tryptophan. Moreover a natural compound produced by cells expressing IDO enhanced pain sensitivity when administered to mice lacking IDO genes. Thus cells expressing IDO caused increased pain sensitivity in infected mice. A distinctive cell type expressed IDO selectively and accumulated in spleens of MuLV-infected mice. Transfer of spleen cells from MuLV-infected mice caused increased pain sensitivity in uninfected mice while eliminating specific cells in MuLV-infected mice abolished enhanced pain sensitivity. Our findings show that host immune responses to virus infections cause increased pain sensitivity and suggest novel ways to alleviate pain during infections.

## Introduction

Enhanced pain sensitivity is a hallmark of inflammation and is a debilitating feature of many clinical diseases, including chronic Human Immunodeficiency Virus-1 (HIV-1) infections [1, 2]. However the underlying causes of chronic pain remain poorly defined [3]. Pain hypersensitivity in rats with inflamed joints correlated with elevated IDO expression in brain, and pain hypersensitivity induced following treatments to induce chronic inflammation did not manifest in mice lacking intact IDO1 genes [4]. These findings were interpreted as evidence that sustained inflammation in tissues outside the central nervous system (CNS) induced IDO activity in brain that was the underlying cause of pain hypersensitivity. However, it is not known how local inflammation in peripheral (non-CNS) tissues induces IDO expression in brain, nor is it clear if IDO mediates pain hypersensitivity in other inflammatory syndromes.

We tested the hypothesis that virus infections enhance pain sensitivity by stimulating IDO using murine models of acute or chronic virus infection in which IDO enzyme activity is elevated. Acute influenza A virus (IAV) infection stimulates robust increase in lung IDO activity which wanes after virus clearance [5]. Murine Leukemia retrovirus (MuLV, LP-BM5 strain) is a natural mouse pathogen that causes persistent infections and pathologies that resemble aspects of human immunodeficiency virus-1 (HIV-1) infections, including sustained IDO activity in lymphoid tissues [6–8]. The role of IDO in MuLV pathogenesis is controversial. A previous study indicated that genetic and pharmacologic IDO ablation led to enhanced interferon type 1 production and increased resistance to MuLV infection [6]. In contrast, a later report found no differences in viral loads and immune pathologies between IDO-sufficient (WT) mice and mice lacking intact IDO1 genes [7]. As MuLV infection also induces peripheral neuropathy and pain hypersensitivity [9, 10] we hypothesized that induced host IDO activity mediates pain hypersensitivity during MuLV infection. We show that IAV and MuLV infections increased pain hypersensitivity via an IDO-dependent mechanism and that a distinctive splenic DC subset expressing the B cell marker CD19 enhanced pain sensitivity in MuLV-infected mice.

## Results

### Acute influenza A infection (IAV) incites pain hypersensitivity by inducing IDO

**Fig 1:**
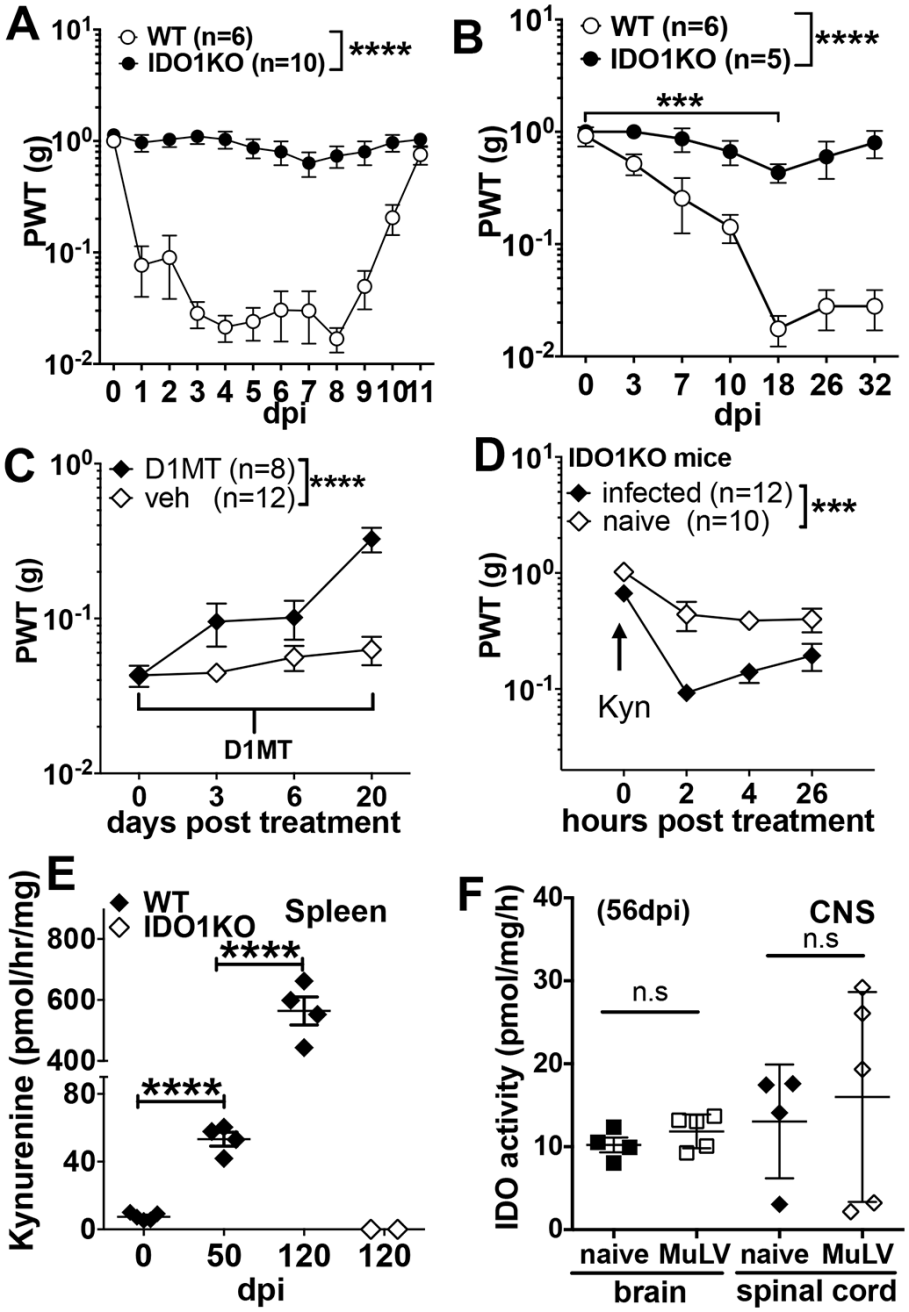
Virus infection enhances pain sensitivity by inducing IDO. **A**. WT (B6) or IDO1KO mice were infected with IAV and the paw withdrawal threshold (PWT) was assessed in infected mice using von Frey filaments at the times indicated post infection (dpi). **B**. WT or IDO1KO mice were infected with MuLV (LP-BM5, i/v) and PWT was assessed. **C**. As in B, except that MuLV-infected WT mice (42dpi) were given drinking water containing D-1MT (2mg/ml) or vehicle. **D.** As in B, except that uninfected (naïve) or MuLV-infected IDO1KO mice were treated with Kyn (200μg. i/v). **E**. IDO activity, expressed as Kyn generated *ex vivo* by homogenized spleen, from MuLV-infected WT and IDO1-KO mice was assessed at the infection times indicated. **F**. IDO activity in brain and spinal cord tissues from naïve and MuLV-infected WT mice (56dpi). **E**. **F.** All experiments were performed twice or more and statistical significance was assessed by 2-way ANOVA with multiple comparison (A-D) or Student’s *t* test (E, F); ****p<0.0001, ***p<0.001, **p<0.01.

IAV respiratory infections in mice stimulate IDO enzyme activity in lungs and lung-draining (mediastinal) lymph nodes [5]. To test if IAV infection incited pain hypersensitivity we assessed mechanical nociception (pain) thresholds by applying mechanical stimuli (von Frey filaments) of increasing force to hind paws until mice responded as described in *Methods*. As soon as one day post infection (dpi) paw withdrawal thresholds (PWT) were reduced significantly, relative to baseline thresholds in the same mice before infection (Fig 1A). Increased pain sensitivity persisted during IAV infection and returned to basal levels 2–3 days after IAV clearance at 7-8dpi [5]. In contrast, no significant change in pain sensitivity manifested during IAV infections in IDO1-deficient (IDO1-KO) mice (Fig 1A). Thus IDO1 genes were necessary to incite pain hypersensitivity during respiratory IAV infections.

### Chronic MuLV infection enhances pain sensitivity by inducing IDO in spleen

MuLV (LP-BM5 strain) causes persistent infections and progressive pathologies, including polyneuropathy and pain hypersensitivity [9–11]. Some features of MuLV infections resemble aspects of clinical HIV-1 infections, including elevated IDO activity in lymphoid tissues [6, 7, 12]. Consistent with previous studies [9, 10], MuLV-infection in B6 (WT) mice bred locally caused progressive increase in pain sensitivity until 18dpi, and levels remained elevated thereafter (Fig 1B and S1A Fig). Increased pain sensitivity also manifested in B6 mice purchased from a commercial supplier (Taconic), though pain sensitivity was slightly less severe and took longer to develop, relative to outcomes in B6 mice bred locally (S1B Fig). Pain sensitivity increased in MuLV-infected IDO1-KO mice relative to naïve mice (Fig 1B and S1B Fig), though responses in IDO1-KO mice were significantly less intense than pain hypersensitivity induced in WT mice. Profound reduction in MuLV-induced pain hypersensitivity did not correlate with major changes in virus titers or host immunopathogenesis (splenomegaly, immunosuppression, cytokine induction) since these parameters were comparable in WT and IDO1-KO mice (S1C–S1F Fig), as reported previously [7]. Thus transient and sustained increase in pain sensitivity manifested during acute IAV or chronic MuLV infections, respectively, and these responses to virus infection depended on IDO1 gene expression but were not linked to changes in virus infection kinetics or host immunopathogenesis.

**Fig 2:**
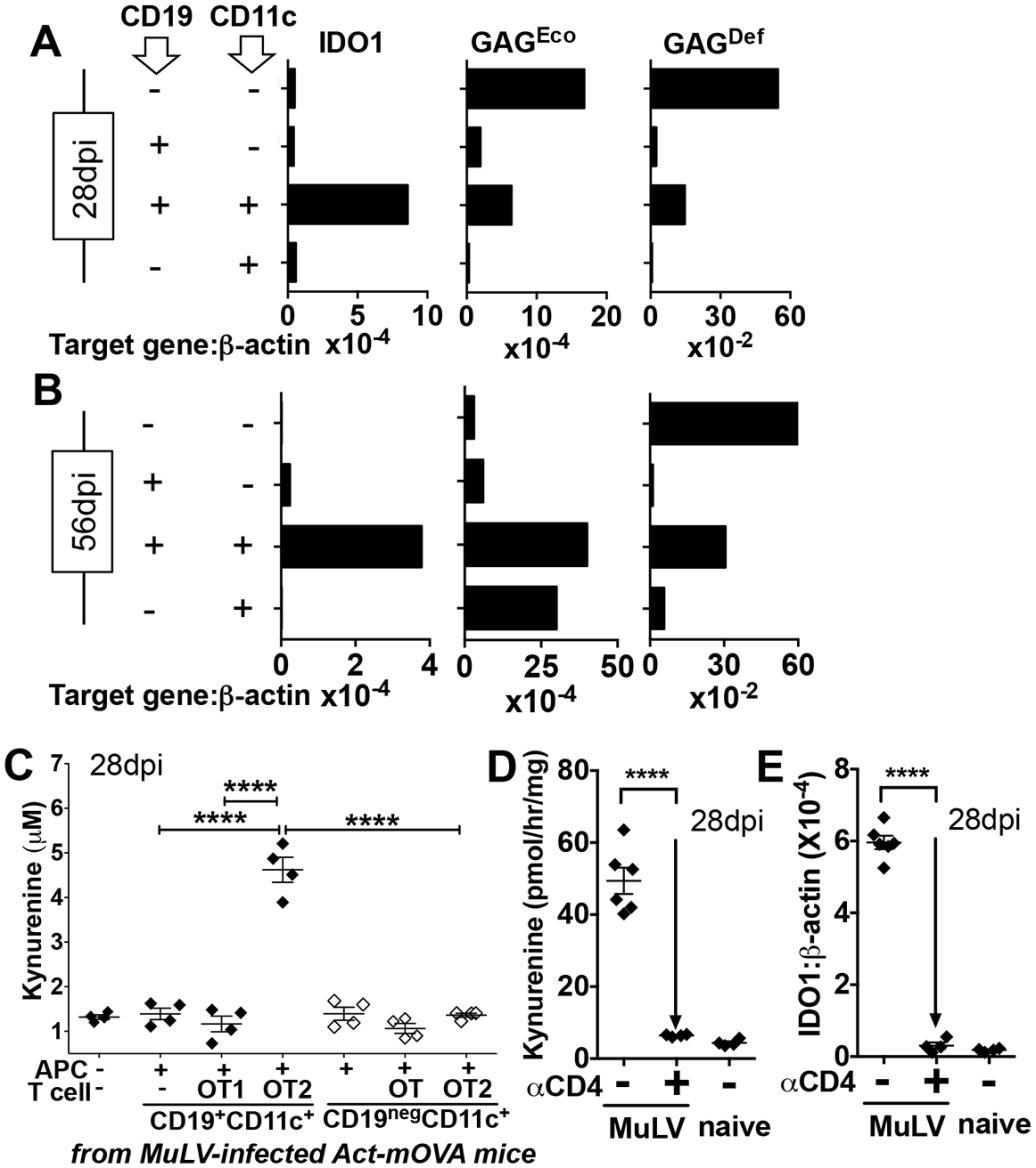
MuLV infection induces selective IDO expression by cells co-expressing DC and B cell markers. **AB.** Splenocytes from MuLV-infected B6 mice at early (A, 28dpi) or later stages (B, 56dpi) of MuLV infection were stained with anti-CD11c and anti-CD19 mAbs, FACS-sorted to select cell populations and RNA prepared for qPCR analyses to detect IDO1, GAG^Eco^ or GAG^Def^ transcripts. **C**. FACS-sorted CD19^+^ or CD19^neg^ DCs (closed or open symbols) from MuLV-infected Act-mOVA transgenic mice were cultured with splenocytes from OT1 or OT2 TCR transgenic mice as indicated. After 72hrs, media was analyzed by HPLC to detect Kyn. Statistical significance was evaluated by ANOVA or Student’s *t* test; ****p<0.0001. **DE**. Anti-CD4 antibody was used to deplete CD4^+^ T cells (28dpi). Spleen IDO enzyme activity (D) and IDO1 transcription (E) were assessed 5 days later by measuring Kyn release and by qPCR. PCR analyses of sorted splenocytes were performed once per time point, coculture experiments were performed 3 times (C), and CD4 depletion experiments were performed twice.

To test if pharmacologic IDO inhibition alleviated pain hypersensitivity the IDO inhibitor 1-methyl-[D]-tryptophan (D-1MT, 2mg/ml) was administered continuously in drinking water to B6 mice with established MuLV infections (60dpi). Oral D-1MT treatment for 20 days led to significant reduction in pain sensitivity, relative to controls given vehicle only (Fig 1C). It is unclear why oral D-1MT treatment did not alleviate pain hypersensitivity more robustly. As spleen IDO activity increased markedly during MuLV infections (Fig 1E) D-1MT may reduce but not abolish spleen IDO activity in this model, though potential off-target effects of D-1MT cannot be excluded. Exposing naïve and MuLV-infected IDO1-KO mice to the natural tryptophan catabolite kynurenine (Kyn, 200μg/mouse, i/v) led to rapid increase in pain sensitivity; this effect was more severe in MuLV-infected than in uninfected (naïve) IDO1-KO mice (Fig 2D), suggesting that Kyn may synergize with cytokines co-induced by MuLV infection to enhance pain sensitivity. Thus cells expressing IDO1 cause pain hypersensitivity in MuLV-infected mice and Kyn released by cells expressing IDO may mediate this response. Consistent with a previous study [6], spleen IDO activity increased progressively during MuLV infection until IDO activity was >100-fold higher at 120dpi (Fig 1E). However IDO activity was undetectable in spleens of MuLV-infected IDO1-KO mice (Fig 1E), indicating that IDO1 genes exclusively encoded MuLV-induced IDO enzyme activity, not IDO2 and tryptophan dioxygenase (TDO) genes encoding enzymes with similar functions. In contrast, at early or late stages of MuLV-infection IDO enzyme activity (Fig 1F) and IDO1 gene transcription in CNS tissues (S2B Fig) were not elevated significantly over basal levels.

### MuLV infection induces a discrete population of dendritic cells to express IDO

To identify cells expressing IDO and MuLV (GAG) genes discrete cell populations were purified by flow cytometry from spleens of MuLV-infected B6 mice and gene transcription was assessed using quantitative RT-PCR. Splenocytes were stained with CD11c and CD19 mAbs since melanoma growth and other inflammatory insults induce selective IDO expression by a discrete subset of dendritic cells (DCs) expressing the B cell marker CD19 [13–16]. At early (28dpi, Fig 2A) and later (56dpi, Fig 2B) stages in MuLV infection when immune pathology partially or fully manifests IDO1 transcripts were detected exclusively in sorted spleen cells expressing CD11c and CD19 (CD19^+^ DCs). Increased IDO1 transcription was not detected in any other spleen cells, including sorted B cells and conventional (CD19^neg^) DCs. At early times in MuLV infection (28dpi), ecotropic helper (GAG^Eco^) and pathogenic (GAG^Def^) MuLV retrovirus genes were transcribed at high levels in sorted cells expressing neither CD19 or CD11c (CD11c^neg^CD19^neg^) and to lesser extents in CD19^+^ DCs, relative to sorted conventional DCs and B cells (Fig 2A). At later stages in infection (56dpi), GAG^Def^ transcription was still relatively high in CD11c^neg^CD19^neg^ and CD19^+^ DCs, though GAG^Eco^ transcription was relatively high in conventional and CD19^+^ DCs but not in CD11c^neg^CD19^neg^ cells (Fig 2B). However, GAG^Def^ and GAG^Eco^ transcription remained relatively low in B cells (CD19^+^CD11c^neg^) at later stages of MuLV. Given previous reports of B cell-specific LP-BM5 expression [17], these findings suggest that LP-BM5 replicates in cells not expressing CD19 or CD11c and in DCs, as well as in B cells. Alternatively, co-selection of (or contamination by) DCs expressing CD19 may explain previous reports of selective MuLV infection in B cells since CD19 and CD11c are commonly used to discriminate between B cells and DCs. Thus CD19^+^ DCs were highly susceptible to MuLV infection and were the only spleen cells induced to express IDO1 during MuLV infection.

To test if increased IDO1 transcription by CD19^+^ DCs led to increased IDO enzyme activity FACS-sorted CD19^+^ and conventional (CD19^neg^) DCs from MuLV-infected Act-mOVA transgenic mice expressing ovalbumin (OVA) in all cells [18] were cultured alone or with splenocytes from (OVA)-specific OT1 (CD8) or OT2 (CD4) TCR transgenic mice and Kyn production was assessed after 3 days. Kyn levels increased significantly in cultures containing sorted CD19^+^ DCs and OT-2 T cells but Kyn was not detected in cultures containing sorted DCs alone, or sorted DCs cultured with OT-1 T cells (Fig 2C). Thus interactions with OT2 T cells were essential for IDO activity to manifest in CD19^+^ DCs from MuLV-infected mice. Consistent with these findings, anti-CD4 mAb treatments to deplete CD4 cells *in vivo* reduced IDO activity (Fig 2D) and IDO1 transcription (Fig 2E) in spleen to basal levels observed in naïve mice. Collectively, these data show that interactions with CD4 T cells is essential for CD19^+^ DCs to express functional IDO in MuLV-infected mice and during culture.

### MuLV infection stimulates DC proliferation and selective maturation of CD19^+^ DCs

**Fig 3:**
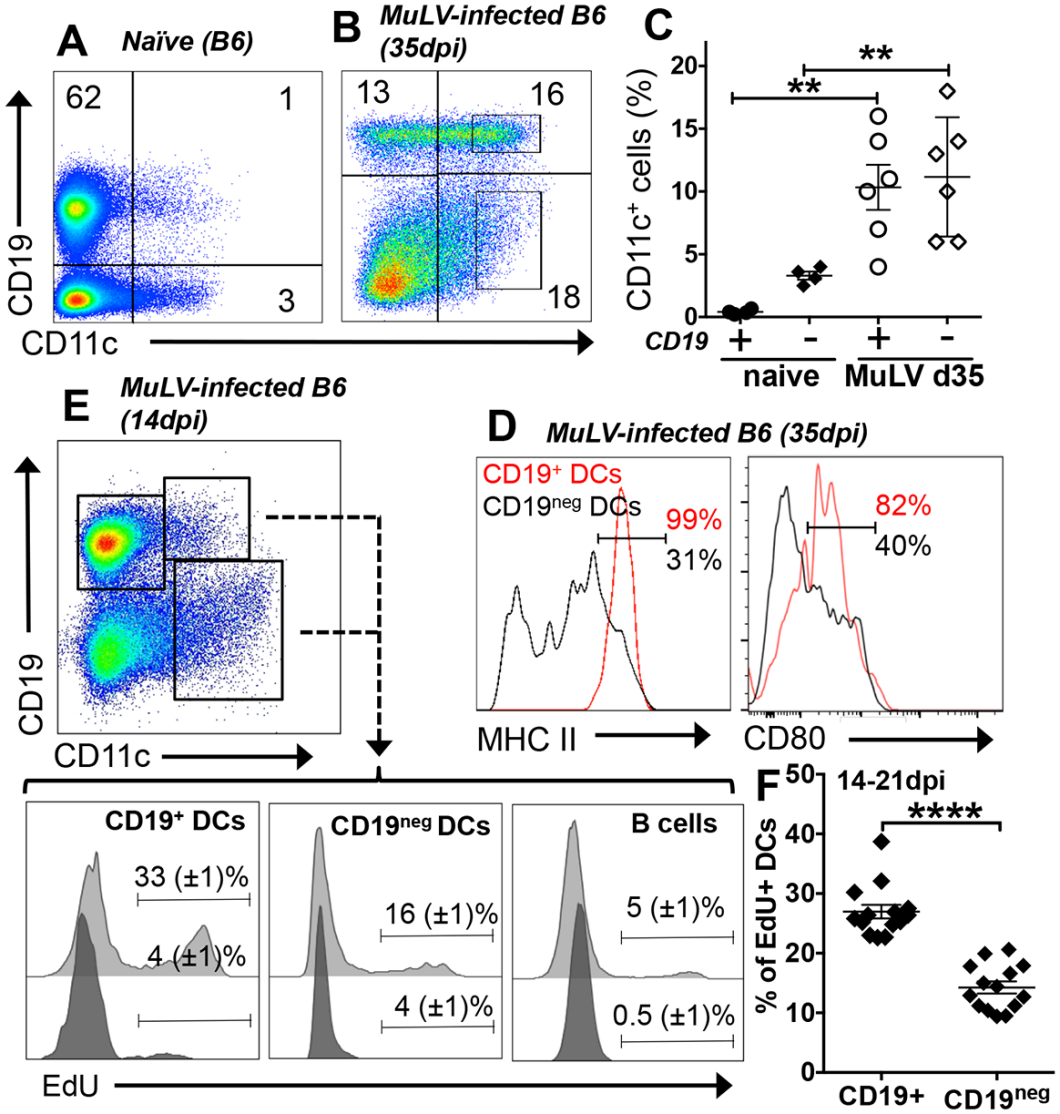
MuLV infection induces selective accumulation, proliferation and maturation of CD19^+^ DCs in spleen. **AB.** Spleens from uninfected (A) and MuLV-infected (B, 28dpi) B6 mice were stained with anti-CD19 and anti-CD11c mAbs and analyzed by flow cytometry. Numbers indicate the percentages of total cells analyzed in each quadrant. **C.** Proportions of CD19^+^ and CD19^neg^ DCs (gated as shown in panel B) expressing CD19 in uninfected (naïve) and MuLV-infected mice. **D**. Flow cytometric analyses of MHCII and CD80 expression by gated CD19^+^ DCs (red lines) and CD19^neg^ DCs (black lines) from MuLV-infected B6 mice (35dpi); markers highlight cells expressing high levels of MHCII or CD80. **E**. Representative FACS plot of proliferating cells in spleens of MuLV infected mice labeled *in vivo*. EdU was injected into MuLV-infected mice (14-21dpi) and spleen cells were analyzed by flow cytometry to detect EdU in gated B cells and DCs 4hrs later. The gating strategy used (dot plot) and markers highlighting EdU-labeled cells are shown for MuLV-infected and naïve (control) B6 mice (light and dark gray histograms, respectively). **F.** Proportions of proliferating cells (EdU^+^) in CD19^+^ and CD19^neg^ DC populations (gated as in panel E). Statistical significance was evaluated by Student’s *t* test; **p<0.01. FACS data are representative of 3 or more experiments.

CD19^+^ DCs are a minor DC population in spleens of naïve mice (Fig 3A, <1% of splenocytes and ~10% of splenic DCs). CD19^+^ DCs expanded substantially after MuLV infection, accounting for ~10% of splenocytes and ~50% of splenic DCs at 35dpi (Fig 3B and 3C). Because MuLV infection induces splenomegaly absolute numbers of CD19^+^ DCs expanded >100-fold relative to numbers in spleens of naïve mice. Conventional (CD19^neg^) DCs also expanded in this period, while relative proportions of splenic B cells were reduced substantially (Fig 3A and 3B). Phenotypic analyses revealed striking differences in maturation between CD19^+^ DCs and conventional DCs in MuLV-infected mice. At 35dpi, CD19^+^ DCs expressed uniformly high levels of MHC class II (MHC II) and CD80 (Fig 3D) characteristic of mature antigen presenting cells (APCs). In contrast, conventional DCs expressed lower and more variable levels of MHC II and CD80/86 (Fig 3D) comparable with levels on immature DCs in naïve mice. Thus CD19^+^ DCs expanded and matured as APCs while conventional DCs also expanded but remained immature during MuLV infection. Higher proportions of splenic DCs stabilized ~28dpi after MuLV infection and *in vivo* labeling with 5-ethynyl-2-deoxyuridine (EdU) at 14-21dpi revealed larger cohorts of dividing (EdU^+^) splenic CD19^+^ (~30%) and conventional (~16%) DCs than B cells (<5%) from MuLV-infected B6 mice (Fig 3E and 3F). *In vivo* treatment with depleting anti-CD4 mAbs reduced EdU incorporation by CD19^+^ and conventional DCs significantly (S3 Fig). Thus MuLV infection induced selective CD19^+^ DC maturation and DC proliferation, as well as selective IDO expression by CD19^+^ DCs dependent on interactions with CD4 T cells.

### Splenic dendritic cells mediate pain hypersensitivity in MuLV-infected mice

**Fig 4:**
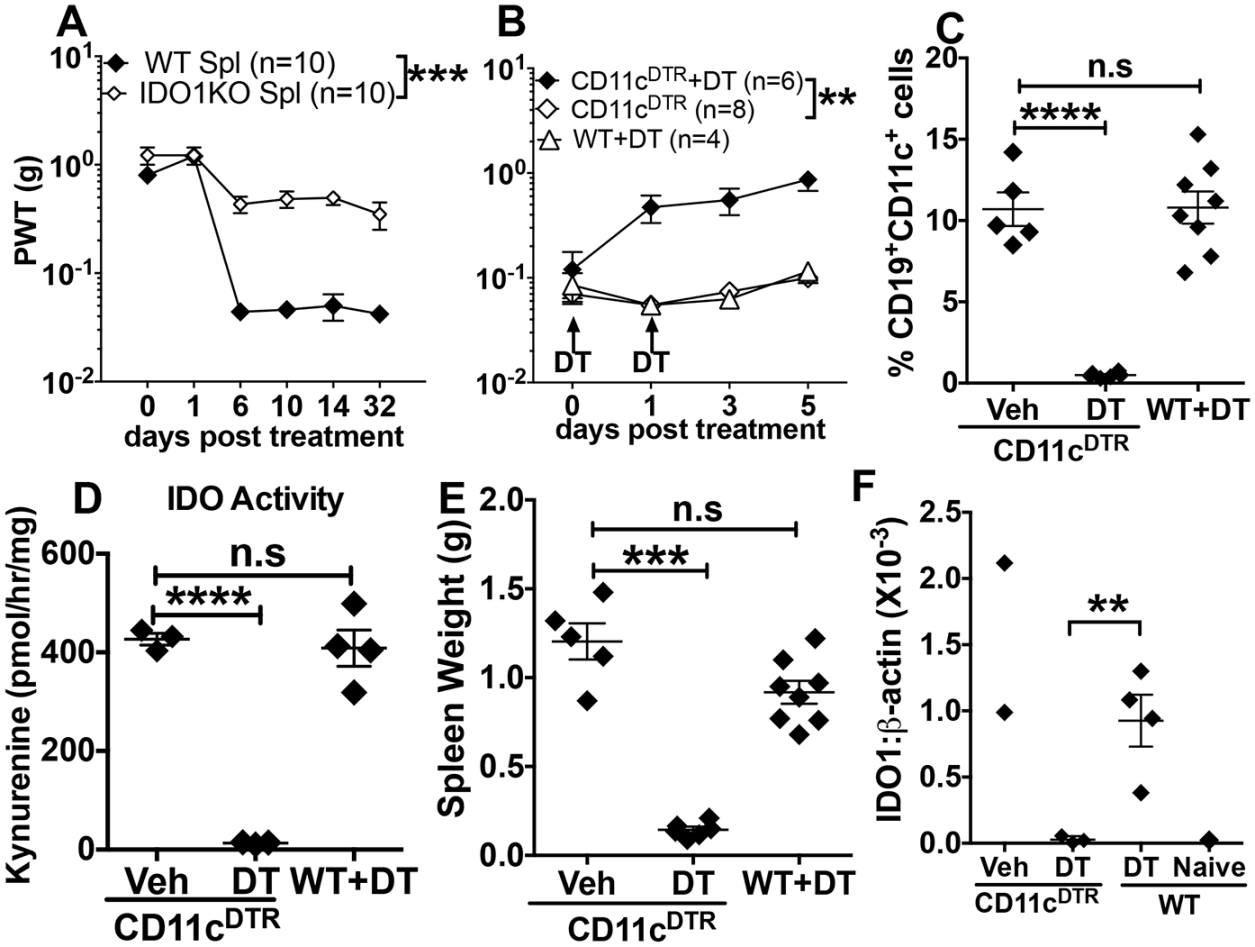
Splenic DCs mediate pain hypersensitivity during MuLV infection. **A.** Naïve IDO1KO mice were exposed to sub-lethal ionizing radiation (3.5Gy) and inoculated with splenocytes (2x10^7^, i/v) from MuLV-infected WT or IDO1KO donor mice 2hrs later. Mechanical nociception (PWT) was evaluated as indicated. **B.** MuLV-infected CD11c^DTR^ mice (56-70dpi) were treated with diphtheria toxin (DT, 100ng on days 0, +2) to deplete DCs and PWT was evaluated. Other CD11c^DTR^ mice were not treated with DT and MuLV-infected WT mice were treated with DT as controls. **C-F**. Analyses of spleen CD19^+^ DCs (C), IDO activity and IDO1 transcription (D and F respectively) and spleen weights (E) in MuLV-infected CD11c^DTR^ mice treated with DT or vehicle (C-F) and in naïve (uninfected) B6 (WT) mice (F). Statistical significance was evaluated using ANOVA or Student’s *t* test; ****p<0.0001, ***p<0.001, **p<0.01. Experiments were performed 2 or more times.

We tested if adoptive transfer of splenocytes from B6 mice with fully established MuLV infections (56-70dpi) enhanced pain hypersensitivity in naïve IDO1-KO recipients. Recipients were sublethally irradiated (3.5Gy) to facilitate donor cell chimerism and six days after transfer of splenocytes from MuLV-infected B6 (WT) donors pain sensitivity was assessed in IDO1-KO recipients. Adoptive transfer of splenocytes from MuLV-infected WT donors caused pain hypersensitivity (Fig 4A), which was sustained until experimental endpoints 32 days after transfer. Splenocyte induced pain hypersensitivity was only slightly less than pain hypersensitivity due to MuLV-infection (Fig 1B). Though splenocytes from MuLV-infected IDO1-KO donors also induced significant increase in pain sensitivity (Fig 4A) these responses were significantly less pronounced than responses to splenocytes from MuLV-infected WT donors; moreover, sublethal irradiation may drive some increase in pain sensitivity. Thus IDO was the major driver of pain sensitivity following splenocyte transfer.

To complement this approach, we tested if *in vivo* DC ablation alleviated pain hypersensitivity using transgenic B6 mice expressing human diphtheria toxin receptor (DTR) under control of DC-specific CD11c gene promoters (CD11c^DTR^ mice). MuLV-infected CD11c^DTR^ mice (56-70dpi) were treated with diphtheria toxin (DT, 10ug/kg, i/p, x2) to ablate DCs and pain thresholds were monitored. DT treatment reduced pain sensitivity rapidly and significantly in MuLV-infected CD11c^DTR^ mice relative to MuLV-infected CD11c^DTR^ mice not exposed to DT and to control naïve WT mice given DT (Fig 4B). As expected, at experimental endpoints (6 days post DT treatment) the proportions of splenic CD19^+^ DCs were reduced significantly in DT-treated, MuLV-infected CD11c^DTR^ mice but DT treatment had no effects on CD19^+^ DCs in MuLV-infected WT (B6) mice (Fig 4C). DT treatment also reduced spleen IDO enzyme activity (Fig 4D) and IDO1 gene transcription (Fig 4F) in MuLV-infected CD11c^DTR^ mice significantly, as levels were comparable to basal levels in naïve (uninfected) mice 6 days after DT treatment. Remarkably, spleen weights were also reduced rapidly and significantly following DT treatment (Fig 4E). In contrast, DT treatment had no significant effects on IDO activity, IDO1 transcription or splenomegaly in MuLV-infected WT (B6) mice (Fig 4D–4F), indicating that the effects of DT treatment were due to ablation of cells expressing DTR. Thus *in vivo* depletion of splenic DCs alleviated pain hypersensitivity in MuLV-infected IDO-sufficient mice and this response was not caused by DT treatment *per se*. Collectively, these data reveal that splenic DCs expressing IDO mediate sustained pain hypersensitivity in MuLV-infected B6 mice.

## Discussion

IDO1 mediated pain and depression in rodents with chronic limb joint inflammation and elevated IDO1 expression in brain hippocampus correlated with these responses [4]. Using the spared nerve injury (SNI) model, Zhou et al. reported that IDO1 expressed in liver mediated depression but did not enhance mechanical pain sensitivity in this model [19]. In the current study we show that acute influenza (IAV) and chronic retroviral (MuLV) infections enhanced mechanical pain sensitivity and that IDO1 ablation alleviated these responses to virus infection.

IAV and MuLV infections stimulate IDO activity in lungs and peripheral lymphoid tissues, respectively. IAV infections induced rapid increase in IDO activity in lung epithelial cells and in DCs located in lung-associated lymph nodes, and IDO activity at these sites returned to basal levels a few days after virus clearance [5]. Pain sensitivity correlated with changed IDO activity during and after IAV infection, consistent with a causative link between IAV-induced IDO activity and pain sensitivity. Similarly, spleen IDO activity correlated with progressive increase in pain sensitivity that peaked before immunopathologies associated with chronic MuLV infections manifested fully. Pain hypersensitivity peaked faster in mice bred locally than in previous reports [9, 10] but onset of peak pain hypersensitivity was slower and comparable with previous studies when mice from a commercial supplier were used, suggesting that mouse husbandry factors influence the kinetics of pain hypersensitivity induced by MuLV infection. This point notwithstanding, IDO1 ablation alleviated pain hypersensitivity almost completely during MuLV infection. Thus IDO activity encoded by IDO1 genes caused acute pain hypersensitivity during IAV infections and was the major factor driving progressive pain hypersensitivity during persistent MuLV infections. Furthermore, IDO2 and tryptophan dioxygenase (TDO) genes encoding enzymes with identical tryptophan catabolizing activities did not compensate for loss of IDO1 genes to enhance pain sensitivity during IAV or MuLV infections. Pro-inflammatory cytokines such as IL-6, TNFα and IL-1β have been reported to enhance pain sensitivity [20]. IDO may mediate or synergize with these effects since IDO is co-induced with pro-inflammatory cytokines in many settings of inflammation because interferons are potent IDO inducers. However, IDO1 ablation was sufficient to block induction of pain hypersensitivity during IAV and MuLV infections, which stimulate production pro-inflammatory cytokine responses.

CD19^+^ DCs were the only cell type induced to express IDO in spleen during MuLV infections and IDO1 expression and enzyme activity were not elevated above basal levels (in naïve mice) in CNS tissues from mice with established MuLV infections. These findings suggested that sustained IDO activity in peripheral lymphoid tissues was sufficient to incite pain hypersensitivity in MuLV-infected mice. Consistent with this interpretation, adoptive transfer of splenocytes from MuLV-infected IDO1-sufficient mice caused pain hypersensitivity in IDO1-deficient recipients, while selective DC depletion *in vivo* alleviated pain hypersensitivity in MuLV-infected IDO1-sufficient mice. While the possibility that IAV and MuLV infections induce IDO activity in CNS tissues to incite pain sensitivity cannot be excluded fully, our findings that splenic DCs from MuLV-infected IDO1-sufficient mice and Kyn enhanced pain sensitivity in IDO1-deficient mice suggest that increased IDO activity in peripheral tissues is sufficient to enhance pain sensitivity. Cells expressing IDO may activate local sensory neurons in peripheral tissues directly or Kyn produced by IDO-expressing cells may act on peripheral or CNS neurons to enhance pain sensitivity. Collectively, our findings support the hypothesis that sustained IDO expression by cells in lungs and splenic CD19^+^ DCs of mice infected with IAV and MuLV, respectively, mediate pain hypersensitivity during infection.

Previously, we reported that splenic CD19^+^ DCs expressed IDO in response to melanoma growth and inflammatory insults that induce interferon type I production, including B7 and TLR9 ligands, DNA nanoparticles and apoptotic cells [13–16, 21, 22]. Moreover, CD19^+^ DCs resemble ‘age-associated B cells (ABCs)’ that accumulate in spleens of aged female mice, in Nba2 mice prone to lupus-like syndromes, and in patients with rheumatoid arthritis [23, 24]. ABC accumulation in aged female mice was TLR7-dependent, suggesting that endogenous retroviral RNA sensing may promote ABC expansion. Similar considerations may explain why CD19^+^ DCs expanded as mature APCs and expressed IDO selectively during MuLV infection. CD19^+^ DCs exhibited potent T cell regulatory phenotypes dependent on IDO and interactions between CD4 T cells and DCs were essential to sustain IDO activity in DCs and to promote Foxp3-lineage regulatory CD4 T cell (Treg) differentiation and activation [25, 26]. Likewise, interactions between CD4 T cells and CD19^+^ DCs were necessary to sustain IDO activity in CD19^+^ DCs from MuLV-infected mice. It is unclear if MuLV antigen-specific interactions between CD19^+^ DCs and CD4 T cells induce IDO activity early in MuLV infection and if these interactions promote Treg differentiation and activation. However exogenous antigens were not required to induce splenic CD19^+^ DCs to express IDO following systemic B7 or TLR9 ligands and DNA nanoparticle treatments [13–15], suggesting that self antigens or antigen-independent pathways induced CD19^+^ DCs to express IDO and activate Tregs *in vivo*. For example, CD4 T cells may produce IFNγ or stimulate innate immune cells to express IFNαβ to induce IDO during MuLV infection. Previously, critical roles for B cells and CD4 T cells in MuLV-induced immune pathogenesis were described [27, 28]. Our findings suggest that CD19^+^ DCs, not conventional B cells, play key roles in MuLV pathogenesis. CD19^+^ DCs are closely related to B cells and express many B cell markers but are a distinct cell lineage with DC attributes [21]. During MuLV-infection CD19^+^ DCs were distinguished from conventional B cells by exhibiting mature APC phenotypes, higher susceptibility to MuLV infection, enhanced proliferation and IDO expression dependent on CD4 T cell interactions.

A previous report described that IDO ablation led to increased plasmacytoid DCs and interferon type I production in response to MuLV infection and to enhanced survival of mice infected with MuLV alone or with MuLV and *Toxoplasma gondii* [6]. In contrast, we found no significant effects of genetic or pharmacologic IDO ablation on the course of MuLV infection or immunopathogenesis, consistent with a previous study by O’Connor and Green using IDO1-KO mice, which also revealed that IDO is not critical, or has a redundant role in regulating host immune responses to MuLV infection [7]. The reason for these disparate outcomes is unclear. Nevertheless, DC depletion led to rapid reduction in splenomegaly, suggesting that DCs may play pivotal and previously unappreciated roles in immunopathologies associated with chronic MuLV infection. However IDO ablation also had little impact on the course of acute IAV infections in mice, though primary CD8 responses were more robust and IAV-specific memory CD8 T cell repertoires differed in the absence of IDO [5]. IDO activity is also elevated in patients with persistent HIV-1 or HTLV1 retrovirus infections, indicating that increased IDO activity is a common response to retroviral infection in mice and humans [12, 29]. It is unclear if IDO contributes to virus control or immunopathogenesis in these clinical syndromes, though previous reports have described human pDC subsets that can express IDO and acquire T cell regulatory phenotypes as a consequence [30, 31]. Despite the lack of evidence supporting a role for IDO in host-virus immune control and immunopathogenesis, the current study reveals that IDO is a major contributory factor driving pain hypersensitivity during acute IAV and chronic MuLV infections. Thus sustained, elevated IDO activity may also contribute to chronic pain associated with acute and persistent clinical infections in humans. If so, inhibition of IDO may help alleviate heightened pain sensitivity induced as a common comorbidity associated with virus infections.

The mechanism by which cells induced to express IDO enhance pain sensitivity during IAV and MuLV infections is unclear. A previous study by Kim and colleagues concluded that increased brain IDO activity mediated pain hypersensitivity in rodent models of experimentally induced arthritis [4]. This conclusion was based on findings that microinjecting IDO inhibitor directly into rat brain hippocampus abolished pain hypersensitivity, while microinjecting IL-6 stimulated brain IDO expression in this rodent arthritis model. Findings from the current study suggest that elevated brain IDO activity may not be necessary to induce pain hypersensitivity during IAV and MuLV infections in mice since lung and lymphoid tissues were the primary sites of elevated IDO activity during IAV and MuLV infections, respectively. Furthermore, increased IDO expression and activity was not detected in CNS tissues of MuLV-infected mice and adoptive transfer of splenocytes from IDO1-sufficient mice with chronic MuLV infections or injection of Kyn heightened pain sensitivity in IDO1-deficient mice. Thus elevated IDO activity in non-CNS tissues was necessary and sufficient to induce pain hypersensitivity during IAV and MuLV infections. It is also unclear how increased IDO activity in lungs or lymphoid tissues leads to elevated sensitivity to mechanical stimulation in hind paws. Though unlikely that limb extremities are impacted directly by IAV and MuLV infection, inflammatory cells expressing IDO or Kyn produced by distal tissues may enter limb extremities or CNS tissues during viral infections and heighten pain sensitivity via direct affects on local nervous tissues.

How increased IDO activity mediates increased pain sensitivity is not understood. IDO catabolizes both serotonin and its precursor tryptophan to generate neuroactive quinolinic (QA) and kynurenic (KA) acids, which mediate diametric neuropathologic and neurodegenerative effects via N-methyl-D-aspartate (NMDA) receptors expressed by neuronal tissues to induce pain and behavioral responses [32]. Unlike QA and KA, which can only cross the blood brain barrier via passive diffusion, Kyn is transported efficiently across the blood brain barrier via large neutral amino acid L-system transporters [33], and may be converted QA and KA to drive neurological responses that enhance pain sensitivity via NMDA receptors expressed in the CNS. Alternatively Kyn generated in non-CNS tissues during IAV or MuLV infections may be converted to QA and KA in non-CNS tissues to promote peripheral neuropathies that heighten pain sensitivity. Kyn is also a weak ligand for aryl hydrocarbon receptors (AhR) expressed by multiple cell types in CNS and non-CNS tissues and modulation of AhR signaling during IAV and MuLV infections may also contribute to peripheral neuropathies that drive increased pain sensitivity. Thus Kyn generated by IDO activity anywhere in the body may potentiate pain sensitivity by interactions of Kyn catabolites with nerve cells in CNS or non-CNS tissues. Interestingly, muscular exercise reduced stress-induced depression by promoting Kyn uptake and catabolism into KA by muscle tissues, thus decreasing the potential of Kyn and its catabolites to cause neurologic effects [34]. This finding suggests that muscular exercise may also to alleviate pain hypersensitivity during infections by reducing Kyn availability.

In summary, acute IAV and chronic MuLV virus infections enhanced pain sensitivity by elevating IDO activity to increase Kyn availability. Links between host inflammatory responses to infection, elevated tryptophan catabolism and increased pain sensitivity suggest that the common co-morbidities of pain and behavioral disorders associated with many progressive inflammatory diseases of clinical significance may arise due to under-appreciated metabolic responses to inflammatory insults such as tumor growth and autoimmunity, as well as infections.

## Materials and Methods

### Mice

B6 mice were purchased from Taconic (Hudson, NY) or bred in a barrier (SPF) facility at GRU. IDO1-KO mice, CD11c^DTR^, OT1 and OT2 TCR transgenic mice were described previously [15, 22].

### Influenza (IAV) infection

IAV A/PR/8/34 (PR8) propagated in embryonated chicken eggs was kindly provided by Ralph Tripp (University of Georgia Athens, GA). Mice were infected with a non-lethal IAV dose (30pfu, 30% of LD50) as described [5].

### MuLV (LP-BM5) infection

SC1/G6 cells infected with MuLV (LP-BM5) were obtained from the NIH AIDS Reagent Program, Division of AIDS, NIAID, NIH [8]. SC1 and XC cells were kind gifts from William Green (Dartmouth). 400ul of SC1/G6 culture supernatant were injected (i/v) to infect mice. Ecotropic (Eco) retrovirus titers in supernatants were determined by XC plaque assay [35]. 1-5x10^4^ pfu Eco retrovirus was injected per mouse [9]. Adoptive transfer of splenocytes from MuLV-infected mice was accomplished by exposing naïve IDO1-KO recipients to sub-lethal radiation (3.5Gy) to create space in hematologic niches.

### Mechanical nociception test

Mechanical nociception was assessed using von Frey filaments (North Coast Medical Inc, Gilroy, CA) to determine paw withdrawal threshold (PWT) as described [36, 37]. In brief, mice were stimulated on both hind paws using a series of von Frey filaments ranging in force from 0.008g to 2g, starting with the 0.008g filament. Positive responses were scored as paw withdrawal occurring two or more times in response to ten successive stimulations. In the event of negative responses, mice were then stimulated with monofilaments of stepwise increasing force. The monofilament that first evoked a positive response was designated the threshold (in grams) and no further monofilaments were applied.

### Flow cytometry

Cells were analyzed on a LSRII flow cytometer (Becton-Dickinson). Data were analyzed using FACS DIVA (BD Bioscience) or FlowJo (Tree Star, Ashland, OR) software. An Aria flow sorter (Becton-Dickinson) was used for sort spleen cells from MuLV-infected mice under BSL2 conditions. Spleen cells from MuLV-infected mice were stained with PE-conjugated rat anti-mouse CD19 (clone 1D3, BD Biosciences) and APC or PECy7-conjugated hamster anti-mouse CD11c (clone N418, eBioScience). Cells were sorted into chilled polypropylene collection tubes (RPMI, 10% FCS) for culture or resuspended (RPMI, 5% FCS) and cell lysis solution was added (Omega Bio-Tek, Norcross, GA) to prepare RNA for analysis. *In vivo* Ethynyl deoxyluridine (EdU) labeling and staining were performed using Click-iT Plus EdU flow cytometry assay kits (Life Technologies) following manufacturer’s instructions with minor modifications. Briefly, 1mg of EdU was injected into each mouse (i/p) and tissues are harvested four hours later. Spleen cells were surface stained with antibodies then fixed and permeablized followed by incubation with fluorophore conjugated azide. Cells are then washed and analyzed on a BD FACS LSRII flow cytometer.

### IDO inhibitor and IDO activity assays

1-methyl-[D]-tryptophan (D-1MT, Indoximod) was kindly provided by NewLink Genetics Inc. D-1MT was administered in sweetened drinking water (2mg/ml) as described [15]. IDO enzyme activity was measured by assessing Kyn produced by cell-free tissue homogenates or present in cell cultures using HPLC as described [5].

### RT-PCR assays

RNA was purified using HP total RNA kits (Omega Bio-Tek, Norcross, GA), reverse-transcribed using a random hexamer cDNA RT kit (Clontech, Mountain View, CA), and quantitative RT-PCR was performed using an iQ5 or CFX system with SsoFast EvaGreen supermix (Bio-Rad, Hercules, CA). Primers for murine β-actin were (forward) 5′-TACGGATGTCAACGTCACAC-3′ and (reverse) 5-AAGAGCTATGAGCTGCCTGA-3′. Validated primers for murine IDO1 were purchased (realtimeprimers.com). Relative expression of GAG^Eco^ and GAG^Def^ were evaluated as described [38]. Threshold cycle (Ct) values were set in the early linear amplification phase; relative expression was calculated as 2^Ct(β-actin) − Ct(target gene)^.

### Statistical analyses

Time courses of mechanical nociception (PWT) were analyzed by two-way ANOVA with multiple comparisons. Unpaired Student *t* tests were used to analyze data generated in all other experiments. Two-tailed *p* values <0.05 were considered significant. GraphPad Prism was used to perform all data analyses.

### Ethics statement

This study was carried out in strict accordance with the recommendations in the Guide for the Care and Use of Laboratory Animals of the National Institutes of Health. All protocols were reviewed and approved by the Animal Care and Use Committee at the Georgia Regents University (AUP#2011–0330).

### IDO1 gene information

Gene ID: 15930; Ensembl: ENSMUSG00000031551; Vega: OTTMUSG00000020648

## Supporting Information

S1 Fig Increased pain sensitivity and inflammatory responses in MuLV-infected mice. **A, B.** Pain thresholds (PWT) were measured at 56dpi and 70dpi in MuLV-infected mice bred in the local facility (A) or purchased from Taconic (B). Slight decreases in pain thresholds in IDO1-KO mice were not statistically significant, relative to pain thresholds in naïve mice. Statistical analyses were performed using Student’s *t* test (A) or 2-way ANOVA (B); *** p<0.001, **** p<0.0001. Data were pooled from 2 or more experiments, except data in panel B was from one experiment. **C-F**. Inflammatory cytokine levels in serum and spleen were measured by multiplex analyses (Luminex) in naïve and MuLV-infected (42dpi) B6 and IDO1-KO mice; IFNγ (C), IL6 (D), TNFα (E) and IP10 (F). Statistical analyses revealed no significant differences in cytokine levels in samples from MuLV-infected mice WT and IDO1-KO mice (Student’s *t* test).
(TIF)

S2 Fig IDO expression in tissues from MuLV-infected mice. Tissues from naïve or MuLV-infected WT mice were analyzed to detect IDO enzyme activity in spleen (A, 56dpi) or IDO1 gene transcripts (qPCR) in spinal cord (B, 30dpi). Statistical analyses were performed using Student’s *t* test; ** p<0.01. Experiments were performed once.
(TIF)

S3 Fig Proliferation of splenic DCs in MuLV-infected mice is driven by interactions with CD4 T cells. MuLV-infected WT mice (28dpi) were treated with anti-CD4 mAbs on two successive days (400μg/mouse, i/v) and were injected with the dye EdU (400 μg/mouse, i/v) 3 days after the second mAb treatment. After 16 hours, spleen samples were stained with CD11c and CD19 mAbs and analyzed by flow cytometry to detect proliferating DCs marked with EdU. Statistical analyses were performed using Student’s t test; **** p<0.0001. Data were pooled from 2 experiments.
(TIF)
