# Supplementary figures and images for: Virus Infections Incite Pain Hypersensitivity by Inducing Indoleamine 2,3 Dioxygenase
Source: PLoS Pathog. 2016 May 11;12(5):e1005615. doi: 10.1371/journal.ppat.1005615 (PMC4863962; doi:10.1371/journal.ppat.1005615)

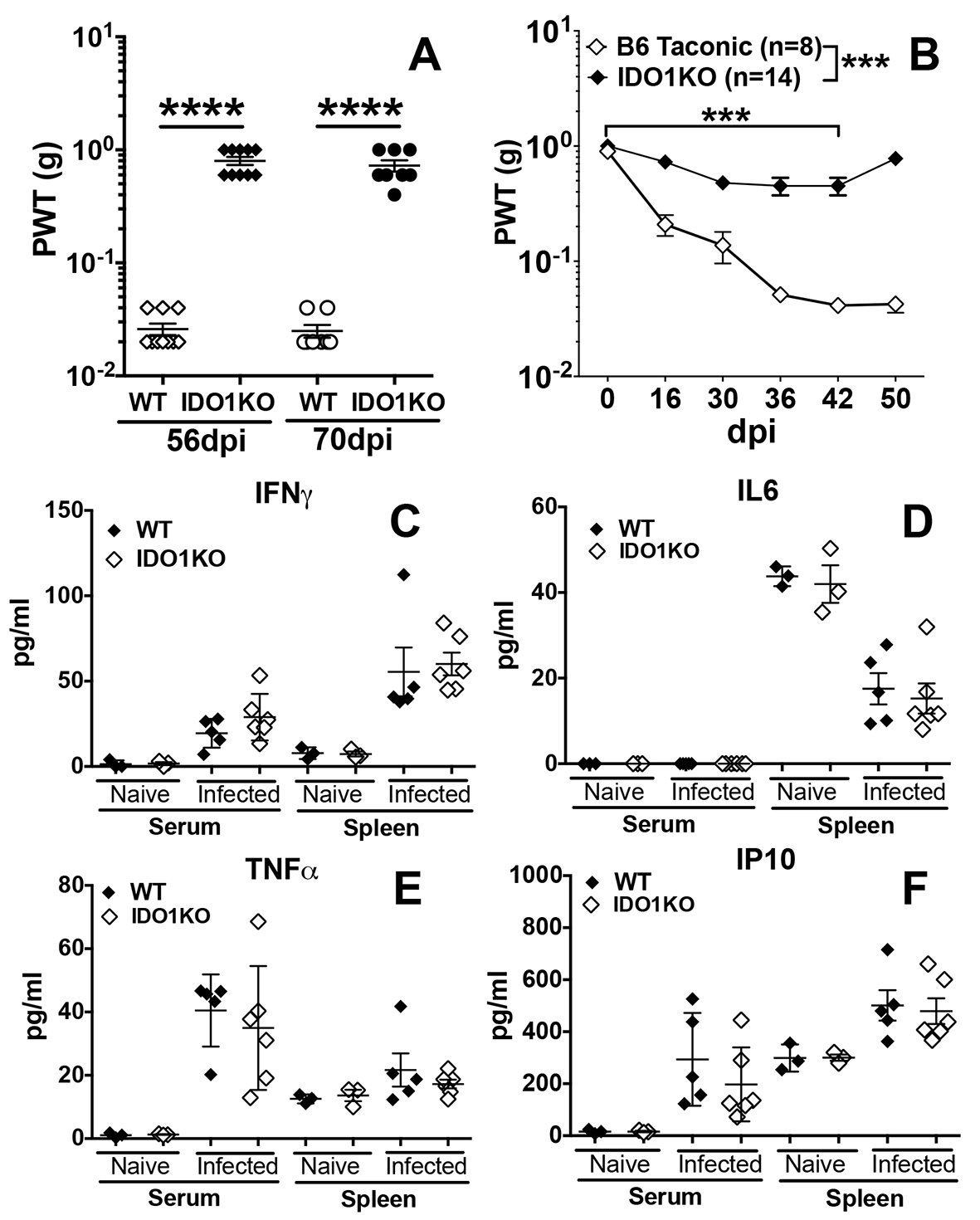

Supplement: S1 Fig — A, B. Pain thresholds (PWT) were measured at 56dpi and 70dpi in MuLV-infected mice bred in the local facility (A) or purchased from Taconic (B). Slight decreases in pain thresholds in IDO1-KO mice were not statistically significant, relative to pain thresholds in naïve mice. Statistical analyses were performed using Student’s t test (A) or 2-way ANOVA (B); *** p<0.001, **** p<0.0001. Data were pooled from 2 or more experiments, except data in panel B was from one experiment. C-F. Inflammatory cytokine levels in serum and spleen were measured by multiplex analyses (Luminex) in naïve and MuLV-infected (42dpi) B6 and IDO1-KO mice; IFNγ (C), IL6 (D), TNFα (E) and IP10 (F). Statistical analyses revealed no significant differences in cytokine levels in samples from MuLV-infected mice WT and IDO1-KO mice (Student’s t test). (TIF) [file ppat.1005615.s001.tif]

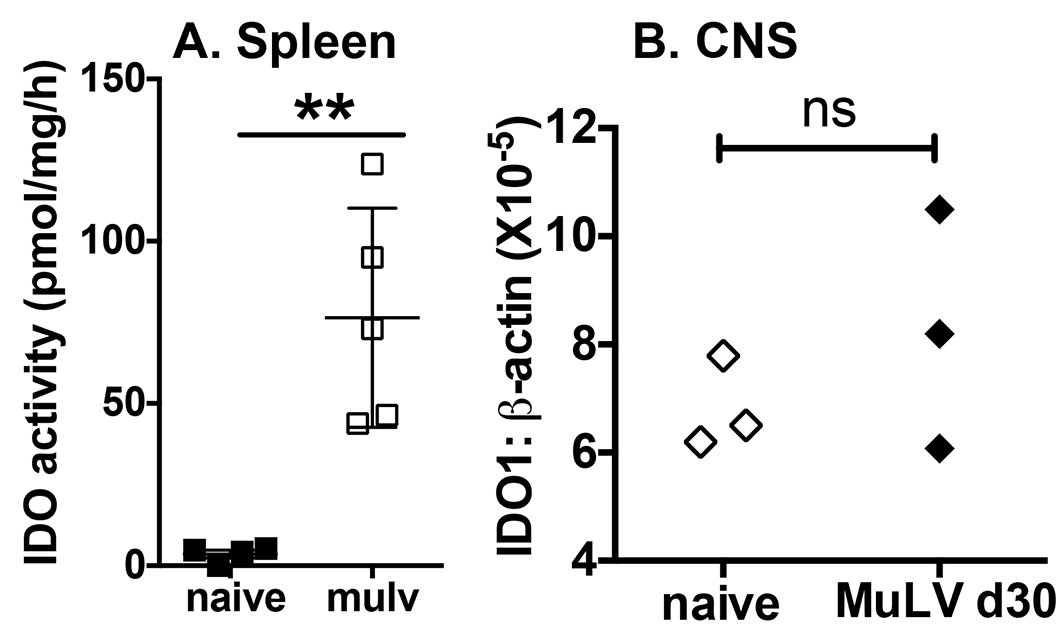

Supplement: S2 Fig — Tissues from naïve or MuLV-infected WT mice were analyzed to detect IDO enzyme activity in spleen (A, 56dpi) or IDO1 gene transcripts (qPCR) in spinal cord (B, 30dpi). Statistical analyses were performed using Student’s t test; ** p<0.01. Experiments were performed once. (TIF) [file ppat.1005615.s002.tif]

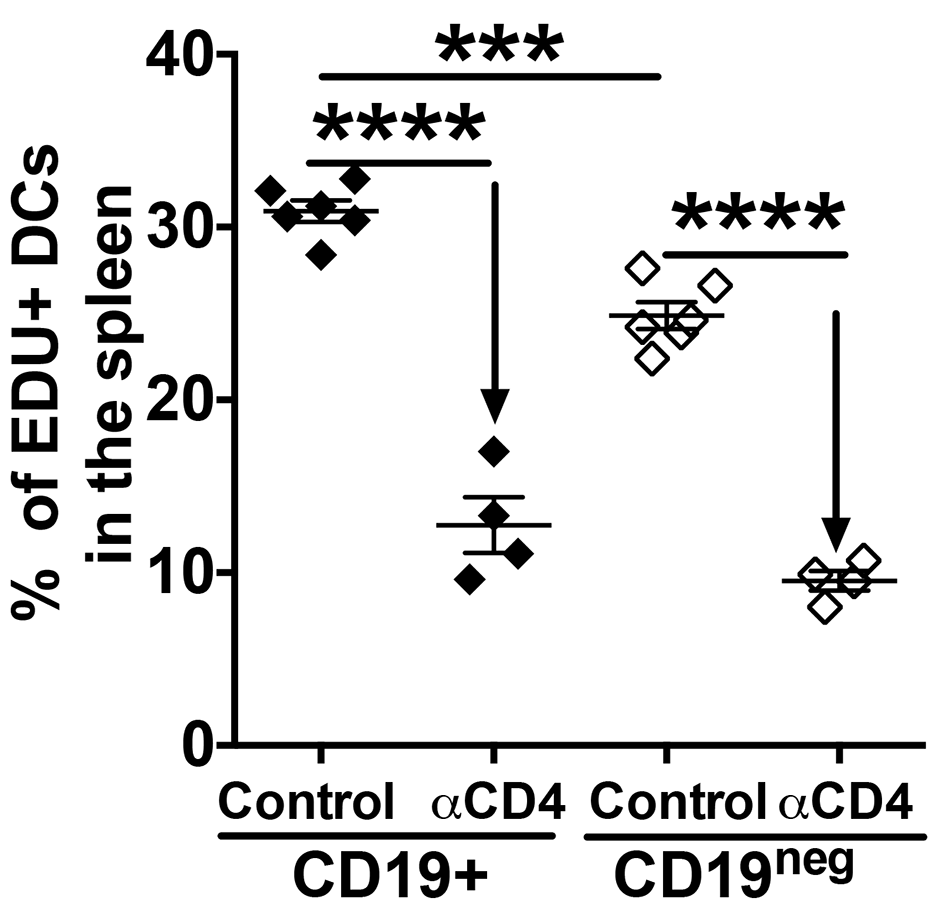

Supplement: S3 Fig — MuLV-infected WT mice (28dpi) were treated with anti-CD4 mAbs on two successive days (400μg/mouse, i/v) and were injected with the dye EdU (400 μg/mouse, i/v) 3 days after the second mAb treatment. After 16 hours, spleen samples were stained with CD11c and CD19 mAbs and analyzed by flow cytometry to detect proliferating DCs marked with EdU. Statistical analyses were performed using Student’s t test; **** p<0.0001. Data were pooled from 2 experiments. (TIF) [file ppat.1005615.s003.tif]
